# The Relationship between Dietary Calcium and Age-Related Macular Degeneration

**DOI:** 10.3390/nu15030671

**Published:** 2023-01-28

**Authors:** Yuan-Yuei Chen, Ying-Jen Chen

**Affiliations:** 1 Department of Pathology, Tri-Service General Hospital, School of Medicine, National Defense Medical Center, Taipei 114, Taiwan; 2 Department of Pathology, Tri-Service General Hospital Songshan Branch, School of Medicine, National Defense Medical Center, Taipei 114, Taiwan; 3 Department of Ophthalmology, Tri-Service General Hospital, School of Medicine, National Defense Medical Center, Taipei 114, Taiwan

**Keywords:** mineral elements, calcium, age-related macular degeneration

## Abstract

Background: Mineral element supplements are widely used in the older adult population. However, little is known of their impact on the progression of age-related macular degeneration (ARMD). The aim of this study was to examine the association between dietary micronutrients and ARMD in older adults. Methods: We enrolled 5227 participants from the National Health and Nutrition Examination Survey (NHANES 2005–2008) in this cross-sectional study. ARMD was evaluated using an ophthalmic digital imaging system and digital camera. Mineral element consumption was collected using a 24-hour dietary recall. The association between mineral element use and the presence of ARMD was determined by multivariable logistic regression. Results: After adjusting for relevant variables, dietary calcium was negatively associated with ARMD (OR: 680, 95%CI: 0.482–0.960). In contrast to dietary form, serum concentration of calcium was not associated with ARMD. Moreover, increased dietary calcium was associated with reduced ARMD (OR: 0.684, 95%CI: 0.468–1.000). Conclusion: A lower consumption of dietary calcium was significantly associated with a higher risk of ARMD. Further longitudinal studies are necessary to explore these findings.

## 1. Introduction

Age-related macular degeneration (ARMD) is one of the most common acquired disorders of the retina that leads to visual loss through non-neovascular and neovascular impairment [1]. ARMD is the third leading cause of visual impairment and blindness in the world and the leading cause of visual loss in developed countries [2]. The prevalence of ARMD in the adult population aged 45 to 85 years is 8.7%, with the number of affected people predicted to be 196 million in 2020 globally [3]. In 2011, the prevalence of ARMD was 7.2 million individuals within the United States, including 5.2 million people older than 60 years [4]. The estimated cost of annual medical care, including inpatient, outpatient, and prescription drug services, for ARMD in the United States was over $4.6 billion [5]. 

The two primary types of ARMD have different causes. Dry form is characterized by dysfunction of retinal pigmented epithelium, photoreceptor loss, and degeneration of retina. Wet form is described by neovascularization of choroid accompanied by hemorrhage, intraretinal leakage, and retinal pigmented epithelium detachments [6]. Most people with ARMD have dry form, which accounts for 85% to 90%. The early and intermediate stage of dry ARMD may be asymptomatic or present with blurred and decreased vision. The late stage of dry ARMD is called geographic atrophy that typically begins in the parafoveal region and involves the central fovea over time [7]. Several potential mechanisms have been reported for the pathogenesis of ARMD. Choroidal ischemia, senescence, vascular endothelial growth factor overexpression, and oxidative damage are suggested to cause choroidal neovascularization [8]. There are several treatment options for ARMD, including laser photocoagulation and vascular endothelial growth factor inhibitors, which, although they may have benefits in limiting vision loss, remain less effective in the long term [9].

In recent decades, many studies were conducted on the impacts of environmental factors on the onset of ocular disorders. Cigarette smoking is considered to increase oxidative stress and reduce antioxidant levels by releasing free radicals that eventually alter vision [10]. Some lifestyle behaviors could be associated with the deterioration of vision [11]. Heavy ethyl alcohol consumption has been reported to be related to cataracts and short-term vision problems [12]. Solid evidence has proposed the effect of supplementary and dietary nutrition with whole foods, macronutrients, and micronutrients on ocular diseases. In a population-based study, greater consumption of calcium and iron was reported to be associated with increased odds of glaucoma [13]. Several nutritional factors are known to play an important role in the development of myopia in childhood [14].

ARMD is a multi-factorial disease involving a continual reaction between genetic and environmental factors [15]. Several risk factors have been identified, including cigarette smoking, numerous genetic variants, obesity, and dietary nutrition [16]. Cigarette smoking is known to contain abundant chemical compounds that have toxic and mutagenic impacts. Its pathological effects through various mechanisms and direct exposure to ocular surface may lead to vascular changes, oxidative damages, and inflammatory process within the cascade of ARMD [17]. Previous studies have reported that overweight is a significant risk factor associated with the progression of ARMD [18]. Accumulating body weight can lead to some physiological changes, such as increased inflammatory cytokines, high level of oxidated stress, and hyperlipidemia, which are involved in the mechanisms of ARMD [19]. Researchers have proposed changes in several genes as possible risk factors for ARMD. Change on the long arm of chromosome 10 in 10q26, which contains two genes, HTRA1 and ARMS2, have been studies to be associated with a possible risk factor of ARMD [20]. Genetic changes in a several complement system genes are also associated with ARMD, such as the CFH gene, lead to an increased risk of developing ARMD [21]. High consumption of vegetables, fish, and fruit is suggested to reduce the risk of ARMD [22]. Low consumption of carotenoids and omega-3 fatty acids is associated with increased progression of ARMD [23]. Antioxidants, vitamins, and some minerals, such as zinc and copper, are suggested to reduce the risk of ARMD [24,25]. The specific micronutrients may have beneficial effects on lowering inflammation and oxidation and decreasing angiogenesis and apoptosis, and therefore, be associated with reduced risk of ARMD [26,27].

To date, the association between dietary micronutrients and the presence of ARMD is not well described. Therefore, the main goal of our study was to examine whether micronutrients had harmful or protective effects on ARMD progression in older adults from the United States using a cross-sectional design.

## 2. Methods

### 2.1. Study Design and Participant Recruitment

Publicly available data of the National Health and Nutrition Examination Survey (NHANES) from 2005 to 2008 was analyzed in this retrospectively cross-sectional study. NHANES is a major program of the National Centers for Health Statistics (NCHS). The nationally representative program engages variable health and nutrition examinations and uses a multistage sampling design that requires a weighting scheme to most precisely estimate the prevalence of diseases in the United States.

**Figure 1:**
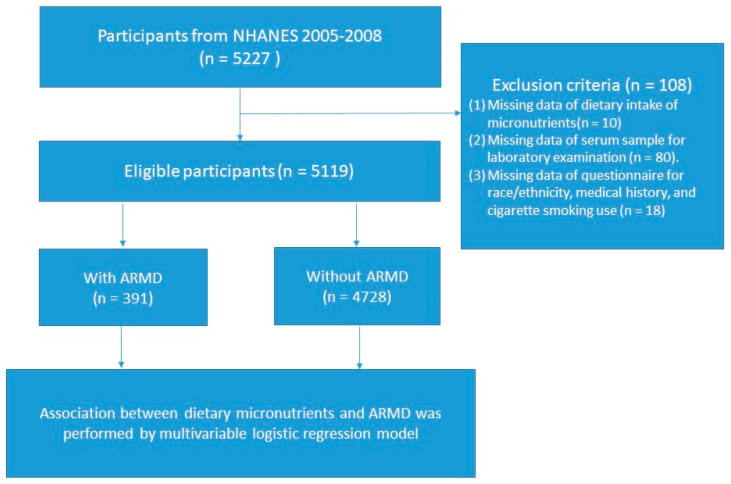
Flowchart of the study.

In total, 5227 eligible participants who had undergone ophthalmological measurement for ARMD were enrolled in this study. Initially, those who had (1) lost data of dietary micronutrients intake (*n* = 10); (2) lost laboratory data of biochemical examination (*n* = 80); and (3) lost epidemiological data, including cigarette smoking use, race, and medical history (*n* = 18), were excluded (Figure 1). Ethical approval was gotten from the Institutional Review Board of the NCHS Ethics Review Board (Protocol #2005–06). All participants provided informed consent before examination and the study design was confirmed following the statement of the Helsinki Declaration. 

### 2.2. Definition of ARMD and Ophthalmic Examination

The assessment of ARMD was done by fundus images and graded by the modified Wisconsin Age-Related Maculopathy Grading Classification Scheme [28,29]. Early ARMD was defined by the presence of numerous small (<63 µm) or intermediate (63–125 µm) drusen. Intermediated ARMD was characterized by either extensive drusen of small or intermediate size, or any large size (≥125 µm) drusen. Late ARMD was characterized by the presence of geographic atrophy or exudative macular degeneration or both. All fundus images were captured by an ophthalmic digital imaging system (CR6–45NM; Canon USA, Inc) and digital camera (EOS 10D; Canon USA, Inc). These images were graded by at least two trained examiners. If the first two examiners did not agree on a diagnosis, a third senior examiner could adjudicate any disagreements.

### 2.3. Assessment of Dietary Mineral Elements

Dietary evaluation and other nutrition assessments, including laboratory biochemical tests, anthropometric measurements, and malnutrition evaluation, are used for nutrition monitoring and developing nutrition policies for population to improve nutritional status [30]. NHANES has been collecting data of dietary intake in several forms since 1970s. NHANES dietary component were merged, forming a consolidated dietary collection database. Numerous experts provided statistical approaches for enhancing accurate estimation of dietary intake and improving usual intakes over the past two decades [31]. The main method for assessing dietary nutrition in NHANES is a 24-hour dietary recall interview. This method is well known for collecting dietary intake in large-scale surveys in the world. Dietary recall interviews are performed in person by well-trained interviewers fluent in English and Spanish. The dietary interview is set in an individual room in the Mobile Examination Center. This room contains a set of measuring standard guides. The 24-hour dietary recall is intended to evaluate accurate detailed information of all foods and beverages consumed over the previous 24 h [32]. To collect more complete pictures of the usual dietary intake, a second 24-hour dietary recall is obtained by a phone call three to ten days later. The decision for continuing with this method over these years in NHANES is based on expert groups consensus [33]. The dietary mineral elements analyzed in our study, including magnesium, copper, zinc, sodium, calcium, and selenium, were recorded using this two 24-hour dietary recall. According to Recommend Dietary Allowance (RDA), the proper value for dietary intake of magnesium, copper, zinc, calcium, and selenium is 420 mg, 900 mcg, 11 mg, 1000 mg, and 55 mcg in men, and 320, 900 mcg, 8 mg, 1200 mg, and 55 mcg in women, respectively [34]. Participants who did not have proper dietary mineral elements were still enrolled in this study. To compare the distinction of serum concentration and dietary intake of calcium, serum calcium levels were also collected in this study. Detailed characteristics of dietary interview procedures are accessible from the NHANES dietary interviewer’s manuals.

### 2.4. Study Variables

In-person interviews were used to collect demographic factors, such as age, gender, race, cigarette smoking status, and previous medical history. The race and ethnicity of participants were classified as Hispanic, non-Hispanic Black, non-Hispanic White, non-Hispanic Asian, and others. Participants were asked “Do you now smoke cigarettes?” to determine cigarette smoking use. Medical history was obtained from a self-reported questionnaire, including coronary heart disease, angina pectoris, and liver condition. Body mass index (BMI) was estimated by dividing the weight (kg) by the square of height (m) (kg/m^2^). Height was estimated using a portal stadiometer, a digital measurement device connected to the acrylic headpiece and interfaced directly with the NHANES Integrated Survey Information System (ISIS) anthropometry application. Weight was estimated using a portal digital weight scale, which was a high-performance instrument linked directly to the ISIS anthropometry application. Serum calcium concentration was analyzed using Beckman Synchron LX20 (Beckman Coulter, Brea, CA). Laboratory biochemical data included glucose, alanine aminotransferase, platelet, creatinine, high density-lipoprotein cholesterol, and C-reactive protein, which were collected by trained medical technologists using standard procedures.

### 2.5. Statistical Analyses

In the current study, all analyses were conducted by the Statistical Package for the Social Sciences, version 22.0 (SPSS Inc., Chicago, IL, USA). Pearson’s chi-square test was used to estimate the significant difference between participants with and without ARMD, the *p*-value of which was defined as ≦ 0.05. Associations between dietary mineral elements, serum concentration of calcium, and odds ratio (OR) for ARMD were calculated by a multivariable logistic regression, which was adjusted by relevant variables. Model 1 was unadjusted. Model 2 was adjusted for age, gender, and race. Model 3 was adjusted for age, gender, race, BMI, and serum laboratory data. Model 4 was adjusted for age, gender, race, BMI, serum laboratory data, past medical history, and cigarette smoking use.

## 3. Results

### 3.1. Study Population

Demographic information of the study population, including 391 participants with ARMD and 4728 participants without ARMD, are presented in Table 1. The mean age was 70.20 ± 11.43 and 58.33 ± 11.99 years in each group, respectively. Participants with ARMD had significantly lower dietary micronutrients, including calcium, magnesium, potassium, and selenium, as well as serum glucose, calcium, and higher serum C-reactive protein (*p* < 0.05).

### 3.2. Association between Micronutrients and ARMD

In Table 2, dietary calcium, dietary magnesium, dietary zinc, dietary sodium, and dietary selenium were significantly associated with ARMD (OR: 0.629, 95% corresponding interval (CI): 0.458–0.853; OR: 0.160, 95%CI: 0.051–0.502; OR: 0.000, 95%CI: 0.000–0.258; OR: 0.858, 95%CI: 0.780–0.944; OR: 0.018, 95%CI: 0.001–0.312) (*p* < 0.001), respectively. After fully adjusting age, gender, race, laboratory data, cigarette smoking use, and past medical history, dietary calcium was still significantly associated with the presence of ARMD (OR: 680, 95%CI: 0.482–0.960).

### 3.3. Comparison of the Relationship between Dietary, Serum Concentration of Calcium, and ARMD

The difference between dietary and serum concentration of calcium and the presence of ARMD is listed in Table 3. We found that dietary calcium was inversely associated with ARMD (OR: 680, 95%CI: 0.482–0.960). In contrast, there was no significant relationship between serum concentration of calcium and ARMD.

### 3.4. Association between Tertiles of Dietary Calcium and The Presence of ARMD

After categorizing dietary calcium into tertiles (Table 4), tertile 2 of dietary calcium had an inverse association with ARMD (OR: 0.647, 95%CI: 0.452–0.928). The relationship was still significant after adjusting for age, gender, race, BMI, serum laboratory data, past medical history, and cigarette smoking use (OR: 0.684, 95%CI: 0.468–1.000).

## 4. Discussion

To date, we investigated the associations between micronutrients and the risk of ARMD among older adults in the United States. Lower consumption of dietary micronutrients, especially calcium, was significantly associated with a higher risk of ARMD. In contrast to dietary intake, serum calcium had no significant association with ARMD. In addition, we found that more than half of participants consumed an insufficient amount of calcium with their diet, which might be a potential hint in the relationship between lower dietary calcium and higher risk of ARMD. There are a few epidemiological studies that have examined the relationship between dietary calcium intake and ARMD. The Australian-based Blue Mountains Eye Study demonstrated lower dietary calcium was associated with higher risk for developing ARMD over a 15 years follow-up [35]. In a population-based study involving a secondary analysis of patients included in the Age-Related Eye Disease Study, increased intakes of supplementary and dietary calcium were related to decreased occurrence of late ARMD [36]. Similar to the Blue Mountains Eye Study and Age-Related Eye Disease Study, our study proposed that increased levels of dietary calcium intake were associated with a decreased occurrence of ARMD in an adult population in the United States. 

Calcium is an important micronutrient involved in a lot of vital physiologic functions. Although studies on the role of calcium primarily focus on bone health, the impacts of dietary calcium and calcium supplement have been oriented towards other health outcomes recently [37]. Dietary calcium was suggested to be associated with blood pression by regulating intracellular calcium in vascular smooth muscle cells [38]. Wang et al. demonstrated that lower levels of dietary intake was significantly associated with a higher risk of cardiovascular mortality in a prospective cohort study [39]. A systematic review reported that higher dietary calcium supplementation was related to lower risk of recurrent colorectal adenomas [40]. Flood et al. indicated a protective effect of dietary calcium on damage of bile-induced colonic mucosa to reduce the risks of colorectal cancer in a female population [41]. Altering calcium homeostasis could cause neurologic damage and trigger cell death, because it has an important role in the regulation and function of neuronal cells [42,43]. Calcium is a second messenger that regulates the most important activities of all eukaryotic cells. It is important to neurons because it is involved in the transmission of the depolarizing signal and leads to synaptic activity by occurring through receptors on plasma membrane and voltage-dependent ion channels [44]. It is said that age-related dysfunction of intracellular organelle membrane and cell membrane may cause neuronal dysfunction to cell damage [45]. Several neurodegenerative disorders are believed to be influenced by calcium dyshomeostasis, such as Alzheimer’s disease, Huntington’s disease, Parkinson’s disease, epilepsy, and brain stroke [46]. Ocular diseases such as glaucoma and macular degeneration were also reported to be associated with the dysfunction of calcium dependent mechanisms [47,48]. Irnaten et al. demonstrated that an elevated calcium concentration was a major driver of fibrosis, which contributed to fibrotic damage in glaucomatous optic neuropathy [49]. Altering intracellular calcium inflow was proposed to contribute to lipofuscin accumulation in retinal pigment epithelial cells, and then cause retinal degeneration [50]. It is reasonable to hypothesize that dietary calcium might play an important role in the progression of macular degeneration with increasing age.

There is accumulating evidence supporting our findings that diet consumption of calcium has a vital role in delaying and preventing the progression of ARMD. Specific micronutrients are reported to have beneficial effects on the progression of ARMD. Aslam et al. proposed potential benefits from vitamin C, vitamin E, carotenoids, and zinc in ARMD by decreasing apoptosis, lowering inflammatory damage, and filtering short wavelength light [51]. Previous studies had clinical evidence suggesting people who ate diets rich in minerals or antioxidant vitamins might have less risk of developing ARMD [52]. Gopinath et al. demonstrated that lower levels of dietary calcium were associated with a higher risk of ARMD progression in a longitudinal study [35]. Higher intakes of dietary and supplementary calcium were proposed to have association with lower risks of ARMD in an adult population [36]. In an animal model study, dietary calcium restriction was reported to enhance exercise-induced oxidative stress by upregulating antioxidant enzymes in rat diaphragm tissue [53]. Hypertension was known as increasing risks of ARMD by aggregating age-related vascular dysfunction or destroying retinal vessels [54]. Nakamura et al. reported that increased dietary calcium intake was shown to lower blood pressure in participants with hypertension. Regular calcium consumption might lead to the prevention and treatment of hypertension and might have a beneficial effect on the progression of ARMD [55].

Drusen are yellow deposits, made up of proteins and lipids, under the retina, and are considered to increase the risk of ARMD. There are two types of drusen associated with ARMD, hard and soft drusen, which have different clinical appearances and different prognoses [56,57,58]. Calcium deposition can be seen in retinal drusens that characterize ARMD [59,60]. However, the relationship between dietary and supplementary calcium and the presence of drusen is unknown. The possible beneficial effect of dietary calcium might be elucidated by other health habits of this population with a low incidence of ARMD. In general, those who have higher dietary or supplementary calcium intakes may have healthier diet habits and may be more inclined to take prescribed medications and exercise regularly [61]. In addition, people with a healthier diet may have more dietary intakes of fish and lutein, which have been reported to be related to a decreased risk of ARMD [62].

Several studies have found relationships between vitamin D deficiency and ARMD. Parekh et al. demonstrated that low serum vitamin D levels were associated with increased risk of early ARMD in a population-based study [63]. Layana et al. proposed that vitamin D deficiency might be an important risk factor of ARMD by mediating inflammation, apoptosis, and oxidative stress [64]. Millen et al. reported that high serum vitamin D concentrations might protect against the development of early ARMD in postmenopausal women [65]. To date, there are limited studies assessing dietary recommendations of vitamin D for prevention of ARMD. Parekh et al. and his colleagues had reported that milk and fish intakes were inversely related to the presence of ARMD [63]. Seddon et al. proposed that higher dietary vitamin D was associated with lower risks of ARMD and smaller size of drusen in a Caucasian male population [66]. In an Asian case control research, Aoki et al. reported that a decreased dietary intake of vitamin D was associated with increased ARMD accompanied by other micronutrients, such as zinc, beta-carotene, and n-3 fatty acid [67]. Vitamin D is an important factor for maintaining calcium homeostasis, which showed to have antiangiogenic and immunomodulatory effects, and may be a plausible role in the progression of ARMD [68]. Dietary calcium is responsible for the modification of vitamin D and serum concentration of parathyroid hormone (PTH) [62]. Patel et al. reported that participants with excessive calcium intakes above the median had lower serum PTH concentrations [69]. In a previous study, low serum PTH levels were suggested to be associated with retinal vascular diameters and a decreased risk of retinal microvascular impairment [70]. Pathogenesis of ARMD has proposed that retinal vascular diameters was associated with the severity of ARMD [71]. Trinh et al. demonstrated that retinal vascular length and vascular diameter were all significantly decreased and narrowed in patients with ARMD [72]. Collectively, we speculated that high dietary calcium might have a protective role in ARMD disease progression. Further studies are needed to investigate the underlying impact of calcium on the physiology of retinal epithelium in ARMD.

This study has some limitations. First, the causal relationship could not be determined in the current study because of the cross-sectional design, even though the relationship between dietary calcium and ARMD were known. Second, institutionalized citizens were not included in the NHANES database; therefore, the prevalence of ARMD might be underestimated. Third, data on dietary supplement intake had missing value inevitably, which was why we did not collect supplementation data. Nonetheless, dietary intake was collected by the recall. Next, methodology about how to calculate the daily intakes of minerals from diet was lacking in the study. Last, the findings of the current study might not be universal because the survey was only collected from a single geographic region. Further studies are necessary to confirm the relationship and modify general recommendations for nutrition.

## 5. Conclusions

In summary, reduced consumption of dietary calcium could lead to an increased risk of ARMD in older adult population of United States. A higher level of dietary calcium may lead to a lower occurrence of ARMD. Our results addressed that the evaluation of dietary mineral elements might be a follow-up parameter in older adults. Further studies are necessary to recognize the potential mechanism in altered calcium homeostasis in the pathogenesis of age-related diseases.

## Figures and Tables

**Table 1:** Characteristics of the study population.

Variables	ARMD (*n* = 391)	No ARMD (*n* = 4728)	*p*-Value
Continuous variables, mean (SD)			
Age (years)	70.20 (11.43)	58.33 (11.99)	0.008
Body mass index (kg/m^2^)	28.51 (5.55)	29.28 (5.58)	0.005
Dietary micronutrients			
Calcium (mg)	801.65 (455.62)	864.34 (535.11)	0.013
Magnesium (mg)	261.18 (116.10)	286.87 (144.44)	0.002
Iron (mg)	14.35 (8.18)	14.85 (8.32)	0.540
Zinc (mg)	10.69 (9.30)	11.64 (11.65)	0.506
Copper (mg)	1.34 (1.09)	1.32 (1.23)	0.176
Sodium (mg)	2844.92 (1425.18)	3209.50 (1713.98)	<0.001
Selenium (mcg)	92.25 (47.49)	103.90 (58.44)	0.003
Laboratory data			
Serum calcium (mg/dL)	8.00 (4.21)	8.60 (5.32)	0.013
Serum glucose (mg/dL)	104.61 (31.33)	106.58 (40.59)	0.010
ALT (U/L)	23.91 (23.01)	25.83 (20.12)	0.351
Platelet (10^3^/uL)	255.71 (72.89)	268.51 (69.37)	0.858
Creatinine (mg/dL)	1.01 (0.34)	0.94 (0.45)	0.208
HDL-Cholesterol (mg/dL)	55.04 (16.36)	53.32 (16.31)	0.493
Cholesterol (mg/dL)	200.45 (41.72)	203.63 (43.08)	0.799
C-reactive protein (mg/dL)	0.57 (1.19)	0.46 (0.85)	<0.001
Category Variables, (%)			
Gender (male)	209 (53.4)	2395 (50.6)	0.338
Non-Hispanic White	283 (72.3)	2532 (53.5)	0.144
Coronary heart disease	40 (10.2)	256 (5.4)	0.659
Angina pectoris	26 (6.6)	184 (3.9)	0.559
Liver condition	15 (3.83)	212 (4.48)	0.569
Cigarette smoking	60 (15.3)	867 (18.3)	0.004

**Table 2:** Association between dietary micronutrients and the presence of age-related macular degeneration.

	Model 1 OR (95% CI)	*p*-Value	Model 2 OR (95% CI)	*p*-Value	Model 3 OR (95% CI)	*p*-Value	Model 4 OR (95% CI)	*p*-Value
*Age-related Macular Degeneration*								
Calcium	0.629 (0.458–0.853)	0.004	0.687 (0.488–0.967)	0.031	0.672 (0.476–0.949)	0.024	0.680 (0.482–0.960)	0.029
Magnesium	0.160 (0.051–0.502)	0.002	0.308 (0.089–1.065)	0.063	0.291 (0.083–1.027)	0.055	0.343 (0.097–1.215)	0.097
Iron	0.000 (0.000–8.454)	0.082	0.000 (0.000–9.542)	0.175	0.000 (0.000–4.356)	0.191	0.000 (0.000–7.307)	0.259
Zinc	0.000 (0.000–0.258)	0.038	0.000 (0.000–7.040)	0.244	0.000 (0.000–6.722)	0.244	0.000 (0.000–4.544)	0.249
Copper	0.000 (0.000–2.595)	0.623	0.000 (0.000–3.330)	0.828	0.000 (0.000–5.315)	0.778	0.000 (0.000–7.310)	0.796
Sodium	0.858 (0.780–0.944)	0.002	0.950 (0.858–1.053)	0.330	0.946 (0.853–1.048)	0.287	0.954 (0.861–1.057)	0.365
Selenium	0.018 (0.001–0.312)	0.006	0.208 (0.010–4.484)	0.316	0.174 (0.008–3.856)	0.269	0.222 (0.010–4.866)	0.339

Adjusted variables: Model 1: unadjusted. Model 2: Model 1 + age, gender, race/ethnicity. Model 3: Model 2 + BMI, serum glucose, ALT, platelet, creatinine, triglycerides, HDL-C, C-reactive protein, hemoglobin. Model 4: Model 3 + history of coronary heart disease, angina pectoris, liver condition, cigarette smoking.

**Table 3:** Association between dietary and serum calcium and the presence of age-related macular degeneration.

	Model 1 OR (95% CI)	*p*-Value	Model 2 OR (95% CI)	*p*-Value	Model 3 OR (95% CI)	*p* Value	Model 4 OR (95% CI)	*p* Value
*Age-related Macular Degeneration*								
Dietary calcium	0.629 (0.458–0.853)	0.004	0.687 (0.488–0.967)	0.031	0.671 (0.475–0.948)	0.024	0.680 (0.482–0.960)	0.029
Serum calcium	0.768 (0.523–1.129)	0.179	0.784 (0.525–1.169)	0.233	0.836 (0.554–1.261)	0.392	0.839 (0.555–1.269)	0.405

Adjusted variables: Model 1: unadjusted. Model 2: Model 1 + age, gender, race/ethnicity. Model 3: Model 2 + BMI, serum glucose, ALT, platelet, creatinine, HDL-C, C-reactive protein, cholesterol. Model 4: Model 3 + history of coronary heart disease, angina pectoris, liver condition, cigarette smoking.

**Table 4:** Association between tertiles of dietary calcium and the presence of age-related macular degeneration.

Dietary Calcium	Model 1 OR (95% CI)	*p* Value	Model 2 OR (95% CI)	*p* Value	Model 3 OR (95% CI)	*p* Value	Model 4 OR (95% CI)	*p* Value
*Age-related Macular Degeneration*								
**T1 vs T3**	0.886 (0.635–1.237)	0.478	0.891 (0.631–1.256)	0.509	0.891 (0.630–1.261)	0.514	0.910 (0.643–1.289)	0.597
**T2 vs T3**	0.647 (0.452–0.928)	0.018	0.696 (0.479–1.012)	0.058	0.680 (0.466–0.992)	0.045	0.684 (0.468–1.000)	0.050

Adjusted variables: Model 1: unadjusted. Model 2: Model 1 + age, gender, race/ethnicity. Model 3: Model 2 + BMI, serum glucose, ALT, platelet, creatinine, HDL-C, C-reactive protein, cholesterol. Model 4: Model 3 + history of coronary heart disease, angina pectoris, liver condition, cigarette smoking.

## Data Availability

The datasets during the current study are not publicly available due to the consent requirement of participants, but sex and age decade-stratified descriptive data are available from the corresponding author upon reasonable request.
